# Immune and Neuroendocrine Trait and State Markers in Psychotic Illness: Decreased Kynurenines Marking Psychotic Exacerbations

**DOI:** 10.3389/fimmu.2019.02971

**Published:** 2020-01-17

**Authors:** Livia De Picker, Erik Fransen, Violette Coppens, Maarten Timmers, Peter de Boer, Herbert Oberacher, Dietmar Fuchs, Robert Verkerk, Bernard Sabbe, Manuel Morrens

**Affiliations:** ^1^Faculty of Medicine and Health Sciences, Collaborative Antwerp Psychiatric Research Institute, University of Antwerp, Antwerp, Belgium; ^2^University Department of Psychiatry, Campus Duffel, Antwerp, Belgium; ^3^StatUa Center for Statistics, University of Antwerp, Antwerp, Belgium; ^4^Janssen Research and Development, Janssen Pharmaceutica N.V., Beerse, Belgium; ^5^Reference Center for Biological Markers of Dementia (BIODEM), Institute Born-Bunge, University of Antwerp, Antwerp, Belgium; ^6^Core Facility Metabolomics, Institute of Legal Medicine, Medical University of Innsbruck, Innsbruck, Austria; ^7^Biocenter, Division of Biological Chemistry, Medical University of Innsbruck, Innsbruck, Austria; ^8^Department of Pharmaceutical Sciences, University of Antwerp, Antwerp, Belgium

**Keywords:** psychosis, schizophrenia, cytokines, kynurenines, kynurenic acid, inflammation, immune, biomarkers

## Abstract

**Objective:** Different patterns of immune system upregulation are present in the acute vs. post-treatment states of psychotic illness. We explored the existence of state and trait markers in the peripheral immune system and two immune-associated neuroendocrine pathways (IDO and GTP-CH1 pathway) in a longitudinal sample of psychosis patients. We also evaluated the association of these markers with neuropsychiatric symptomatology.

**Method:** Plasma concentrations of peripheral blood markers were measured in a transdiagnostic group of 49 inpatients with acute psychosis and 52 matched healthy control subjects. Samples were obtained in patients within 48 h after hospital admission for an acute psychotic episode (before initiation of antipsychotics), after 1–2 weeks and again after 8 weeks of treatment. Kynurenine, kynurenic acid (KA), 3-hydroxykynurenine (3-HK), quinolinic acid (QA), phenylalanine, tyrosine, nitrite, and neopterin were measured using HPLC and LC-MS/MS analysis. Concentrations of CRP, CCL2 (MCP1) and cytokines were determined with multiplex immunoassay. PANSS interviews and cognitive tests were performed at baseline and follow-up. Mixed model analyses were used to identify trait and state markers.

**Results:** Patients had significantly higher plasma concentrations of CRP, CCL2, IL1RA, and lower concentrations of KA and KA/Kyn at all time points (F7.5–17.5, all *p* < 0.001). Increased concentrations of IL6, IL8, IL1RA, TNFα, and CCL2 and decreased QA and 3-HK (F8.7–21.0, all *p* < 0.005) were found in the acute psychotic state and normalized after treatment. Low nitrite concentrations at admission rose sharply after initiation of antipsychotic medication (F42.4, *p* < 0.001). PANSS positive scale scores during the acute episode correlated with pro-inflammatory immune markers (*r* ≥ |0.5|), while negative scale scores correlated inversely with IDO pathway markers (*r* ≥ |0.4|). Normalization of KA and 3-HK levels between admission and follow-up corresponded to a larger improvement of negative symptoms (*r* = 0.5, *p* < 0.030) A reverse association was found between relative improvement of SDST scores and decreasing KA levels (*r* = 0.5, *p* < 0.010).

**Conclusion:** The acute psychotic state is marked by state-specific increases of immune markers and decreases in peripheral IDO pathway markers. Increased CRP, CCL2, and IL1RA, and decreased KA and KA/Kyn are trait markers of psychotic illness.

## Introduction

**Figure 1:**
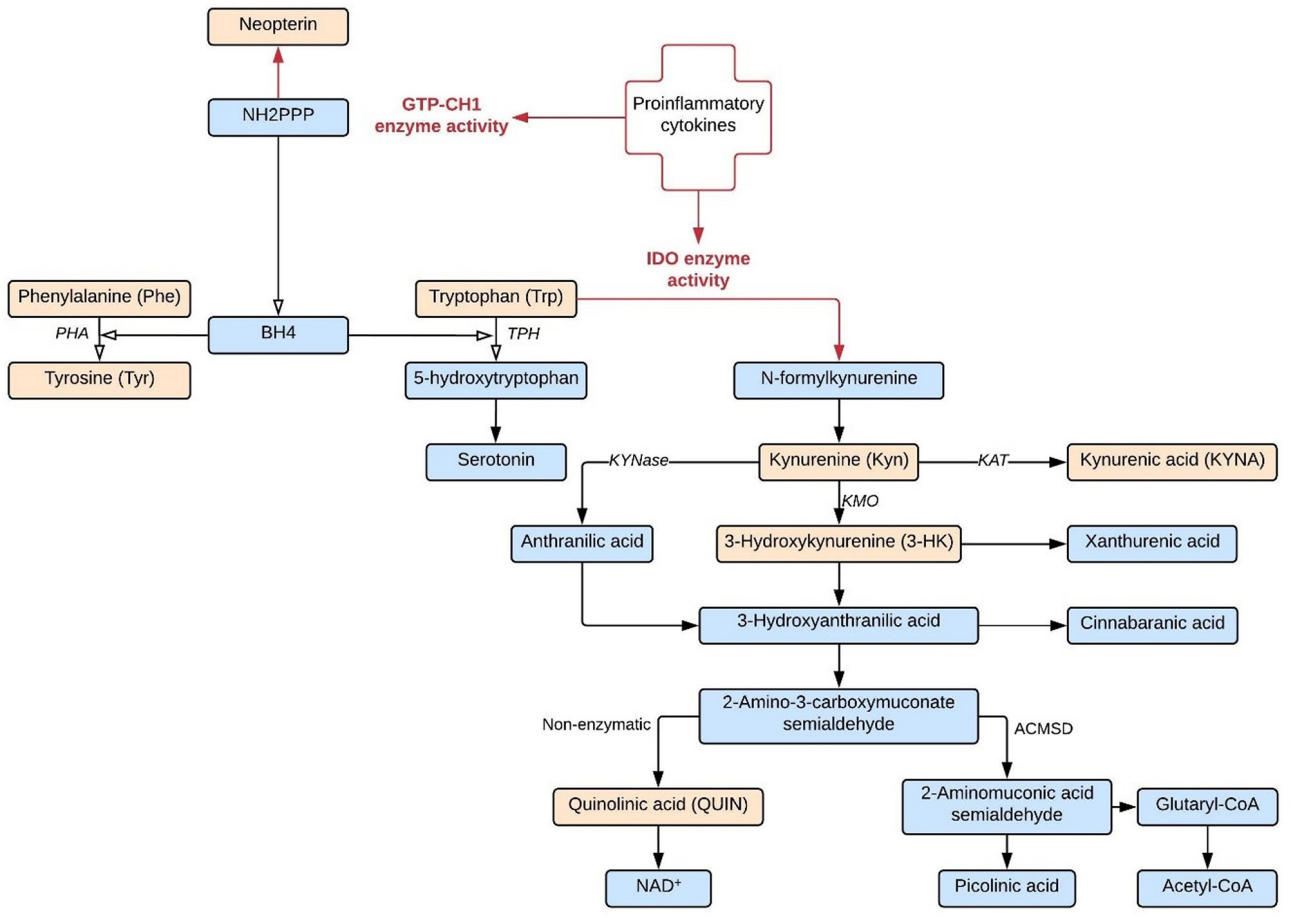
IDO and GTP-CH1 neuroendocrine pathways. Both IDO and GTP-CH1 neuroendocrine pathways are upregulated by proinflammatory cytokines. Biological compounds indicated in orange are included as biomarkers in this study.

Premorbid dysregulation of the immune system has been identified as an important factor of vulnerability for schizophrenia (1–3). Immune system activation could also be involved in schizophrenia symptom development by upregulating two neuroendocrine pathways which affect the biological availability of the two main monoamine neurotransmitter precursors: tryptophan (Trp) and tyrosine (Tyr) (cfr. Figure 1).

Stimulation by inflammatory molecules, particularly IFNγ, strongly activates indoleamine-2,3-dioxygenase (IDO) and the closely related tryptophan 2,3-dioxygenase 2 (TDO2), the first and rate-limiting enzymes of tryptophan breakdown into kynurenine (Kyn). In particular IDO has been linked to immune functioning: the enzyme is found in a variety of immune cells, including microglia in the central nervous system (CNS), and is often upregulated when the immune response is activated (4). Kynurenine is further degraded into downstream metabolites such as 3-hydroxykynurenine (3-HK), quinolinic acid (QA), and kynurenic acid (KA), which directly affect neuronal functioning. KA is an endogenous antagonist of all ionotropic excitatory amino acid receptor activities and therefore considered a protective metabolite against neurotoxic NMDA receptor agonist QA (5). However, abnormal accumulation of KA could lead to glutamatergic hypofunctioning and induce psychotomimetic effects (6, 7). In animal models, elevated KA levels are associated with sensory gating deficits and schizophrenia-like cognitive dysfunctions (e.g., deficits in set-shifting tasks, spatial working memory, hippocampal long-term potentiation, and attentional processing of environmental stimuli) (8–12).

A second neuroendocrine pathway which is upregulated by proinflammatory cytokines runs through GTP cyclohydrolase 1 (GTP-CH1), producing neopterin and tetrahydrobiopterin (BH4). BH4 is an essential cofactor of phenylalanine-hydroxylase (PHA), tyrosine-hydroxylase, tryptophan-hydroxylase, and nitric oxide synthases (NOS) and plays a fundamental role in the synthesis of monoamine neurotransmitters (13). Neopterin is released by activated human monocytic cells at the expense of BH4 activity. BH4 is particularly sensitive to oxidative stress and BH4 deficiencies have been reported in patients afflicted with various chronic inflammatory conditions as well as schizophrenia (14–16). In summary, the IDO and GTP-CH1 pathways may represent a neuroendocrine link between the immune system abnormalities and neuropsychiatric symptoms of psychosis patients.

Meta-analyses have confirmed peripheral changes in the levels of cytokines, chemokines, lymphocytes, and oxidative stress markers of patients with schizophrenia during acute exacerbations (acute psychotic relapse or first psychotic episode), which normalize with antipsychotic treatment (summarized in De Picker et al.) (17). These “state” markers are differentiated from other “trait” markers that remain significantly altered throughout the disorder (18, 19). Thus, different patterns of immune system upregulation are present in the acute vs. post-treatment states of psychotic illness. We hypothesized similar state-dependent changes to exist in the immune-associated neuroendocrine pathways.

The aim of this study was to identify *state* and *trait* IDO and GTP-CH1 pathway markers together with immune system markers in a longitudinal sample of patients during acute psychotic exacerbation, and to evaluate the association of these markers with neuropsychiatric symptomatology.

## Materials and Methods

### Participants

We recruited a transdiagnostic group of 49 inpatients fulfilling the Diagnostic and Statistical Manual of Mental Disorders (DSM−5) criteria for a diagnosis within the spectrum of primary psychotic illnesses (DSM-5 #295.1–295.6, 295.9, 298.9). Patients were newly admitted to one of three major psychiatric hospitals in the Antwerp region of Belgium (University Psychiatric Hospital Antwerp Campus Duffel, Multiversum Campus Alexianen, and Campus Amedeus) for first-episode psychosis or for acute relapse of psychosis, as defined by Positive and Negative Syndrome Scale (PANSS) interview scores (20), and were antipsychotics-naïve or–free for at least 4 weeks prior to hospital admission. Additionally, 52 healthy age-, gender-, and BMI-matched controls from the same area were enrolled. All controls were considered healthy based on clinical evaluation with vital signs and laboratory tests (including liver enzymes, hematology, HBV, HCV, and HIV serology, and urinalysis). Individuals with a personal medical history of (1) auto-immune disorders or any chronic or recent acute physical illnesses associated with abnormal immune changes or who used anti-inflammatory or immunomodulating drugs or systemic corticosteroids within the last 3 weeks; or (2) substance use disorders according to DSM-5 criteria (except nicotine or caffeine) within the last 3 months were excluded. Control subjects with a personal history of any psychiatric disorders or family history (first degree relatives) of psychotic or bipolar disorders were also excluded.

Patients' symptom severity was measured using the Positive and Negative Syndrome Scale (PANSS). Psychotic exacerbations were defined by a total score of ≥14 on the positive scale of the PANSS and at least a score of 5 on one item or a score of 4 on two “psychotic” PANSS items P1, P3, P5, or G9 at Screening (20, 21). PANSS interviews were conducted with patients during the acute psychotic state and at follow-up (within 1 week of each blood sampling) by a trained interviewer. Together with the interviews, cognitive measures (Symbol-Digit Substitution Task Test and WAIS IV Letter-Number Sequencing Task) were obtained.

The study procedures were described in detail to all participants, who gave written informed consent. The local ethics committees of University Hospital Antwerp, Emmaüs, Brothers of Charity, and Spes et Fides approved the study.

### Blood Sampling

Non-fasting blood samples were obtained from all participants in a standardized manner at three different occasions in patients and two occasions in controls. Whenever possible, a first (*Unmedicated Psychosis; UMP*) sample was obtained from patients within 48 h of hospital admission (available for 37 of 49 patients), after which antipsychotic medication was initiated as determined by clinical needs. Subsequently, a sample was obtained during the first 2 weeks of hospitalization (*Psychosis*, in some patients this represented the first study sample; mean 10.2 ± 5.6 days after *UMP* sample) together with the first PANSS interview and cognitive tests. Finally, the last (*Follow-up*) sample was obtained after at least 8 weeks of treatment (mean 81.2 ± 25.9 days after *Psychosis* sample) together with the second PANSS interview and cognitive tests. In controls, two samples were drawn at the same time of day at least 6 weeks apart (mean 77.1 ± 43.2 days). Blood samples were collected from January 2014 to May 2016 without any dietary or fasting protocols and drawn via a forearm vein in EDTA and citrate containing tubes. See Supplementary Figure 1 for timing of blood draws. Blood samples were transferred to the laboratory (<30 min) on cold packs and centrifuged for 10 min at 4°C immediately after arrival. The resulting plasma was aliquoted into Eppendorf tubes which were frozen immediately at −80°C and kept frozen until analysis.

### LCMS Quantitative Analysis of Trp, Kyn, QA, and KA by LC-MS/MS

IDO pathway analytes Trp, Kyn, QA, and KA were measured in citrate plasma samples using liquid chromatography–tandem mass spectrometry (LCMS) analysis at the Institute of Legal Medicine and Core Facility Metabolomics of the Medical University of Innsbruck, Austria as described elsewhere (22). Samples were shipped frozen to Innsbruck where they were stored at −20°C until analysis. Samples were processed in batches of 20–30 samples. Additionally, two quality control samples were analyzed with each batch of patient samples added to each batch. These plasma samples were kindly donated by the blood bank of the Medical University of Innsbruck. They were stored at −20°C prior to use. The order in which the samples were processed was pre-specified to make sure all samples belonging to the same participant were in the same batch, and each batch contained a similar number of patient and control samples (see also Supplementary Material).

### HPLC Quantitative Analysis of Phe, Tyr, Nitrite, and Neopterin

Free concentrations of phenylalanine (Phe), Tyr, nitrite, and neopterin were measured in EDTA plasma samples by high performance liquid chromatography (HPLC) analysis at the Center of Chemistry & Biomedicine of the Medical University of Innsbruck, Austria as described elsewhere (4, 13). For an estimate of NO production, the stable NO metabolite nitrite (NO2^−^) was determined in the cell-free culture supernatants by the Griess reaction assay (Promega, Madison, Wisconsin).

Plasma aliquots were shipped frozen to Innsbruck at two different timepoints (6 months interval) and were stored at −20°C until analysis. They were processed in batches of 20–30 samples with a pre-specified order, as above.

### HPLC Quantitative Analysis of 3-HK

3-hydroxykynurenine (3-HK) was measured at the University of Antwerp Department of Pharmaceutical Sciences by HPLC with electrochemical detection as described elsewhere (23). Two hundred milliliters of citrate plasma sample was deproteinized with 40 ml of 0.23 M perchloric acid. To 120 ml of deproteinized sample was added a solution of 20 g/l sodium decane sulphonate and 1 g/l EDTA in acetonitrile:water (40:60) and injected into a HPLC system equipped with a Chromolith Performance 3.0 × 100 mm column with a Chromolith guard cartridge. Elution solvent was 2.0 g/l decane sulphonic acid, 100 mg EDTA, and 5.9 ml phosphoric acid in 1,250 ml of water and 130 ml ACN. pH is brought to 3.5 with trimethylamine. Flow was 1.7 ml/min. Detection was coulometrically using an ESA electrochemical detector at 350 mV. Recovery of 3-HK was more than 95%. Within-assay CV was 4.7%, between-assay CV was 14.7%. Specificity was checked by observing retention by changing solvent composition.

### Quantitative Immunoassays

Immune markers of interest were measured in duplicate in EDTA plasma by an electrochemiluminescence immunoassay technique developed by Mesoscale Discovery (Rockville, USA), according to the manufacturer's instructions. We used standardized kits V-PLEX Proinflammatory Panel 1 Human Kit (for detection of IFNγ, IL10, IL12p70, IL1B, IL6, IL8, and TNFα), V-PLEX Cytokine Panel 1 Human Kit (for IL17A), V-PLEX Chemokine Panel 1 Human Kit (for monocyte chemoattractant protein-1, MCP1/CCL2) and V-PLEX Vascular Injury Panel 2 Human Kit (for C-reactive protein, CRP). Additionally, IL-1RA was detected using a custom 4-Spot Prototype Human IL-1RA kit. Concentrations for each cytokine were calculated by fitting the sample signals on a 4-parametric logistic calibration curve. Assays were excluded if concentrations were below detection threshold in >50% of participants (as was the case for IFNγ, IL10, IL12p70, IL17A), as well as all data points with an intra-assay coefficient of variation >15% (cfr. Table 2).

### Statistical Analysis

To estimate the activity of PHA, the ratio of the substrate Phe vs. the concentrations of the enzyme product Tyr (Phe/Tyr) was calculated. A similar ratio was calculated for Kyn/Trp and KA/Kyn as indices of IDO and KAT, respectively.

All statistical analyses were performed in JMP version 13 and Review Manager 5.3. Non-normally distributed markers were log normalized prior to the use of parametric statistics (IL1RA, IL6, IL8, CCL2, TNFα, CRP, QA, QA/KA). We applied a reflected transformation to the distribution of neopterin because of negative skew. Outlier plasma concentrations of markers (>3 × z-score) were excluded from analysis.

Baseline differences in clinical and demographic parameters between cohorts were examined by two-tailed independent *t*-tests for continuous variables and Pearson chi-square test for categorical variables. Pearson correlation analyses tested the association between different markers and symptom severity. All medium-to-high strength correlations (*r* > |0.3|) are reported. All data are presented as mean ± SD unless otherwise indicated.

**Figure 2:**
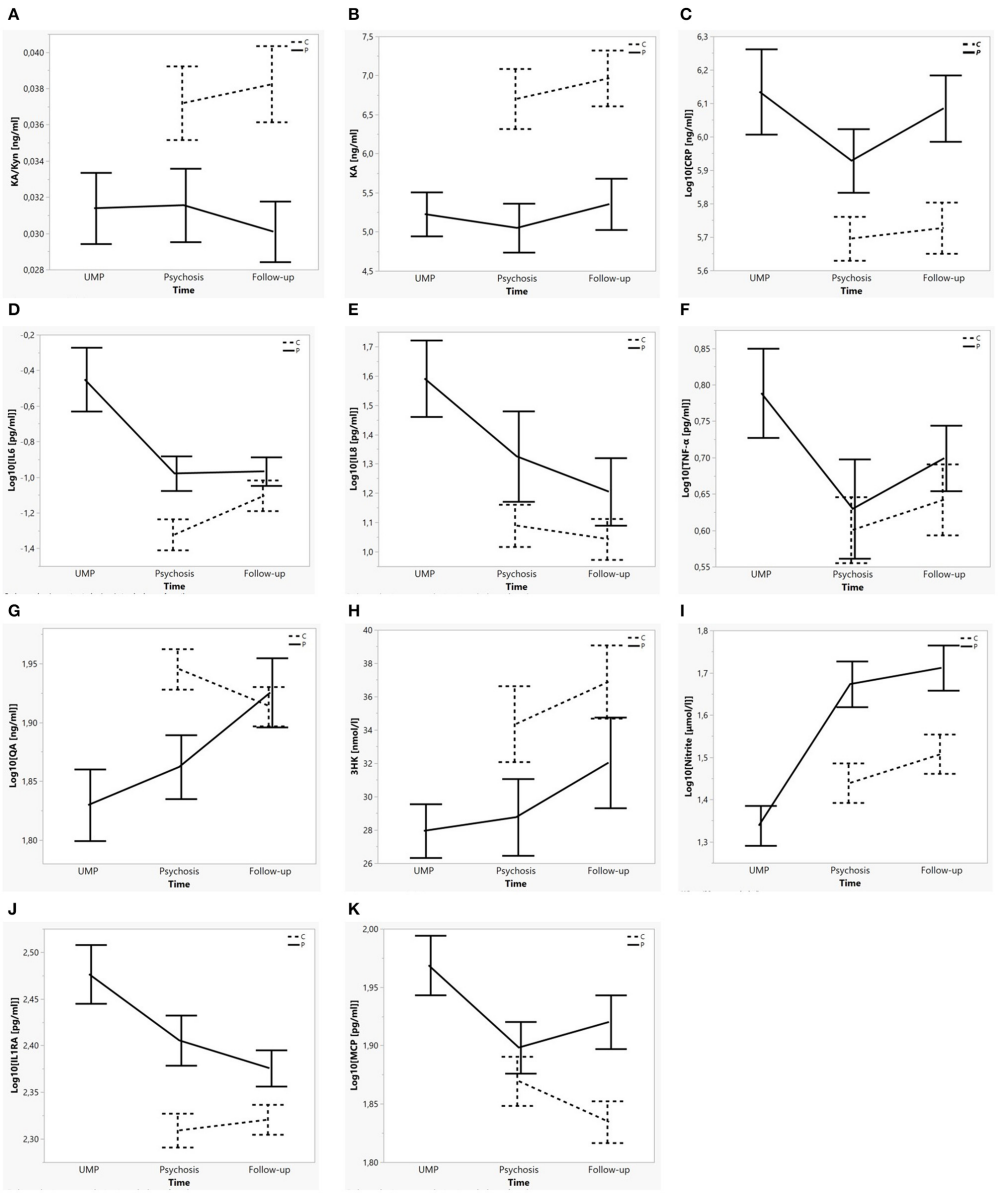
Mean concentration of each biological marker per cohort over time. **(A–C)** Trait markers for psychotic illness: KA/Kyn, KA, CRP. **(D–I)** State markers for the acute psychotic state: IL6, IL8, TNFα, QA, 3HK, Nitrite. **(J,K)** Trait ánd state markers for psychotic illness and the acute psychotic state: IL1RA, CCL2/MCP1. Error bars represent mean ± SE. Dotted lines indicate control subjects, full lines patients. “UMP” (unmedicated psychosis) represents the first blood sampling in patients; “Psychosis” represents the second blood sampling in patients and the first blood sampling in controls; “Follow-up” represents the last timepoint in patients and controls.

A series of linear mixed model restricted maximum likelihood (REML) analyses were performed with the different immune and neuroendocrine markers as the dependent variables. To model trait differences in peripheral immune and neuroendocrine markers between the cohorts as well as differences related to the acute psychotic state vs. post-treatment state in patients, Subject was included as random effect and Cohort, State (i.e., the psychotic state, including both UMP, and Psychosis timepoints) nested in Cohort and Batch as fixed effects (marker = [SubjID] + Cohort + State[Cohort] + Batch). Because the graphical presentation of the identified markers (demonstrated in Figure 2) indicated distinct results for the samples taken at admission (during Unmedicated Psychosis; UMP), the above linear mixed model for each marker was repeated using UMP as State. Dependent variables which demonstrated significant effects for State [Cohort] in either of two mixed models were considered *state* markers, whereas variables for which Cohort was significant in both mixed model analyses were considered *trait* markers. Subsequently, the models were adjusted for sex, age, BMI, and smoking.

All significance levels are reported as two-sided *P*-values, corrected for multiple testing using the Benjamini-Hochberg implementation (24) of the False Discovery Rate (FDR) correction. An FDR adjusted *p*-value of 0.05 was used as cutoff for significance (significant *p*-values are indicated by ^*^).

## Results

### Demographics

We enrolled 52 controls and 49 patients, of whom seven dropped out of the study prior to the last timepoint. The first unmedicated UMP blood sample was available for 37 out of 49 patients. Demographic information and patients' PANSS scores are presented in Table 1. PANSS scores were not available for analysis in three patients. Patients' baseline PANSS total scores, positive subscale and general psychopathology subscale scores were significantly higher than at follow-up.

**Table 1:** Demographics, PANSS interview, and cognitive test results at baseline and follow-up.

	**Patients**	**Controls**	**Test statistic**
*N*	49	52	
Age range	19–49 years	18–47 years	
Age mean + SD	32.4 ± 7.5 years	28.5 ± 7.0 years	t2.66, df99, *p* = 0.010
M:F (absolute)	42:7	39:13	
M:F (pct)	86:14	75:25	χ1.82, *p* = 0.177
BMI mean + SD	24.9 ± 4.0	23.9 ± 3.7	t1.29, *p* = 0.201
Smoking	63.8%	5.9%	χ36.77, *p* < 0.001
THC use prior to study *(heavy use defined as more than three times per week)*	18.8% light 20.8% heavy	3.9% light	χ19.5, *p* < 0.001
Duration of illness	6.1 ± 5.9 years		
1st episode	26.1%		
	**Acute psychosis (*****n*** **=** **46)**	**At follow-up (*****n*** **=** **39)**	
PANSS total score	83.4 ± 14.6	59.1 ± 12.1	t10.9, df37, *p* < 0.001
PANSS positive scale score	24.8 ± 5.0	10.8 ± 2.9	t17.9, df37, *p* < 0.001
PANSS negative scale score	18.4 ± 7.4	17.2 ± 7.0	t1.3, df37, *p* = 0.197
PANSS general psychopathology scale score	40.6 ± 7.9	30.9 ± 7.1	t6.9, df37, *p* < 0.001
SDST score (numbers correct)	44.0 ± 12.0	48.0 ± 11.7	t2.84, df37, *p* = 0.008
LNS adjusted score	5.0 ± 2.2	5.2 ± 2.4	t0.77, df37, *p* = 0.446

*Data are expressed as mean values ± SD*.

### Trait and State Markers of Psychosis

Increased concentrations of IL6, IL8, TNFα, CCL2, and decreased Nitrite were identified as markers of the unmedicated acute psychotic state [State(Cohort) for UMP: all *F* = 8.68–42.36; *p* = ≤ 0.001–0.004), whereas increased IL1RA (*F* = 8.33; *p* = 0.005) and decreased 3-HK (*F* = 12.81; *p* = 0.001) and QA (*F* = 16.07; *p* < 0.001) were state markers of the whole acute psychotic episode, both before and after initiation of antipsychotic medication. We identified increased IL1RA, CRP, and CCL2 and decreased KA and KA/Kyn as trait markers (Cohort: all *F* = 5.96–17.39; *p* = ≤ 0.001–0.017) (see Figure 2). No significant differences were identified for Trp, Phe, Tyr, Phe/Tyr, and Neopterin. Results of the analyses are summarized in Table 2 and Supplementary Table 1.

**Table 2:** Concentrations of different immune markers at the different timepoints.

**Compound**	**UMP (at admission)**	**Psychosis (<2 weeks in hospital)**	**Follow-up (>8 weeks treatment)**	**Control**	**Datapoints excluded or missing**
IL6 (pg/ml)	3.09 ± 6.83	0.38 ± 1.12	0.20 ± 0.21	0.16 ± 0.26	*N = 11*
IL8 (ng/ml)	0.98 ± 4.17	1.30 ± 4.61	0.20 ± 0.77	0.03 ± 0.08	*N = 4*
IL1RA (ng/ml)	0.33 ± 0.16	0.28 ± 0.15	0.25 ± 0.07	0.22 ± 0.07	*N = 16*
IL1B (pg/ml)	0.07 ± 0.29	0.03 ± 0.08	0.02 ± 0.08	0.27 ± 1.86	*N = 25*
TNFα (pg/ml)	8.01 ± 5.55	6.23 ± 5.51	6.35 ± 6.41	5.44 ± 4.02	*N = 41*
CRP (μg/ml)	4.03 ± 6.22	2.18 ± 3.10	2.20 ± 1.99	1.00 ± 1.22	*N = 4*
CCL2 (pg/ml)	99.12 ± 36.96	82.99 ± 24.80	88.85 ± 36.06	75.17 ± 27.46	*N = 3*
Trp (μg/ml)	10.68 ± 2.66	10.57 ± 2.19	10.21 ± 1.93	10.88 ± 1.76	*N = 0*
Kyn (ng/ml)	172.30 ± 36.58	165.64 ± 40.45	179.38 ± 43.41	185.16 ± 47.09	*N = 0*
KA (ng/ml)	5.22 ± 1.62	5.05 ± 2.03	5.35 ± 1.99	6.83 ± 2.66	*N = 0*
QA (ng/ml)	72.39 ± 24.79	78.55 ± 30.59	91.43 ± 38.88	88.39 ± 25.17	*N = 0*
3-HK (nmol/l)	27.95 ± 8.98	28.77 ± 14.55	32.04 ± 16.09	35.69 ± 15.53	*N = 16*
Nitrite	26.16 ± 15.86	59.86 ± 34.151	63.64 ± 34.25	39.47 ± 30.16	*N = 6*
Neopterin	5.80 ± 2.07	5.44 ± 2.31	6.50 ± 5.15	5.56 ± 2.74	*N = 6*

*Data are expressed as mean values ± SD. Phe and Tyr are not represented due to significant batch effects*.

### Adjustment for Confounders

The above analyses were adjusted for the effects of sex, BMI, smoking, and age: (1) A significant interaction between cohort and sex existed in KA (*F* = 17.39; *p* < 0.001) and KA/Kyn (*F* = 13.64; *p* < 0.001), with male controls demonstrating higher KA and male patients demonstrating lower KA; (2) Nitrite levels were lower in men compared to women in both cohorts (*F* = 12.0, *p* < 0.001); (3) Higher BMI significantly increased concentrations of CRP, IL1RA, and QA in both cohorts; (4) The effect of smoking was not significant; (5) A significant interaction between cohort and age was found for IL1B (*F* = 7.96; *p* = 0.006) and Kyn/Trp (*F* = 8.94; *p* = 0.004) and a significant main effect of age for IL6 (*F* = 7.45; *p* = 0.008). Results are summarized in Supplementary Table 1.

### Relationship Between Immune and Neuroendocrine Markers

TNFα concentrations in patients but not controls correlated with Kyn (*r* = 0.310–0.509), Kyn/Trp (*r* = 0.361–0.451), QA (*r* = 0.432–0.481), and QA/KA (*r* = 0.349–0.518), but not with KA at each of the three timepoints. In contrast, in controls CRP and IL1RA concentrations correlated with IDO pathway metabolites. Results are summarized in Supplementary Table 2.

### Relationship With Clinical Symptoms and Patient Characteristics

PANSS positive scale scores during the acute psychotic state correlated with concentrations of IL1B (*r* = 0.463, *p* = 0.026), IL6 (*r* = 0.541, *p* = 0.008), and CRP (*r* = 0.507, *p* = 0.014) and correlated inversely with Neopterin (*r* = −0.427, *p* = 0.021), whereas PANSS negative scale scores correlated with Kyn (*r* = 0.458, *p* = 0.013), QA/KA (*r* = 0.379, *p* = 0.040) and inversely with KA/Kyn (*r* = −0.400, *p* = 0.032). Furthermore, the relative change in PANSS negative scale scores between the acute and post-treatment states [(acute–post-treatment)/acute] correlated inversely with the relative change in KA (*r* = −0.470, *p* = 0.024) as well as 3-HK (*r* = −0.465, *p* = 0.026).

Both groups improved between the first and second rounds of cognitive testing but patients performed significantly poorer compared to controls (SDST controls 70.0 ± 12.1, vs. patients 45.6 ± 12.1, within-pairs *F* = 0.76, *p* = 0.386, among-pairs *F* = 85.0, *p* < 0.001; LNS controls 9.96 ± 2.9 vs. patients 5.10 ± 2.3, within-pairs *F* = 5.6, *p* = 0.020, among-pairs *F* = 85.4, *p* < 0.001). The relative change in SDST performance over time in patients [(acute–post-treatment)/acute] correlated inversely with the relative change in KA (*r* = −0.489, *p* = 0.010) and KA/Kyn (*r* = −0.358, *p* = 0.067).

## Discussion

### Trait and State Markers of Psychotic Illness

In the present study, we tested the hypothesis that state or trait increases in levels of pro-inflammatory cytokines may be accompanied by state-specific IDO and GTP-CH1 pathway abnormalities in schizophrenia spectrum disorders. We defined five immune (IL1RA, IL6, IL8, TNF, CCL2) and three neuroendocrine (QA, 3-HK, Nitrite) *state* markers of acute exacerbations which normalized after treatment with antipsychotics. Five *trait* markers (IL1RA, CRP, CCL2, KA, KA/Kyn) differentiated between patients and controls at any of the timepoints. IL1RA and CCL2 were both state and trait markers, distinguishing patients from controls as well as patients during a psychotic episode from those at post-treatment follow-up. Kyn/Trp was a trait marker only in participants older than 30 years of age.

Our findings confirm the association of schizophrenia and the acute psychotic state with increased peripheral (pro-inflammatory) immune markers, as described earlier by Miller et al. in two meta-analyses of blood cytokine and CRP levels. Miller et al. identified IL1B, IL6, and TGFβ as state markers for acute exacerbations, while IL12, IFNγ, TNFα, sIL2R, and CRP were trait markers. IFNγ and IL12p70 were measured in our study but did not meet quality control criteria required for further analysis. CCL2 was also identified as a trait marker (defined here as elevated in both first- and multiple-episode schizophrenia patients irrespective of treatment) in another recent meta-analysis (25).

However, although our patients exhibited state and trait immune activation, the GTP-CH1 pathway did not differentiate patients from controls (except for nitrite) and the IDO pathway appeared overall downregulated in patients vs. controls. Although this finding contradicts our original hypothesis of immune-activated IDO upregulation, it is in line with results from two recent amino-acid profiling studies (one cross-sectional study in 208 first episode psychosis patients and one 7-month follow-up study in 38 schizophrenia patients) in which tryptophan and kynurenine were decreased in participants with schizophrenia vs. controls (26, 27).

**Figure 3:**
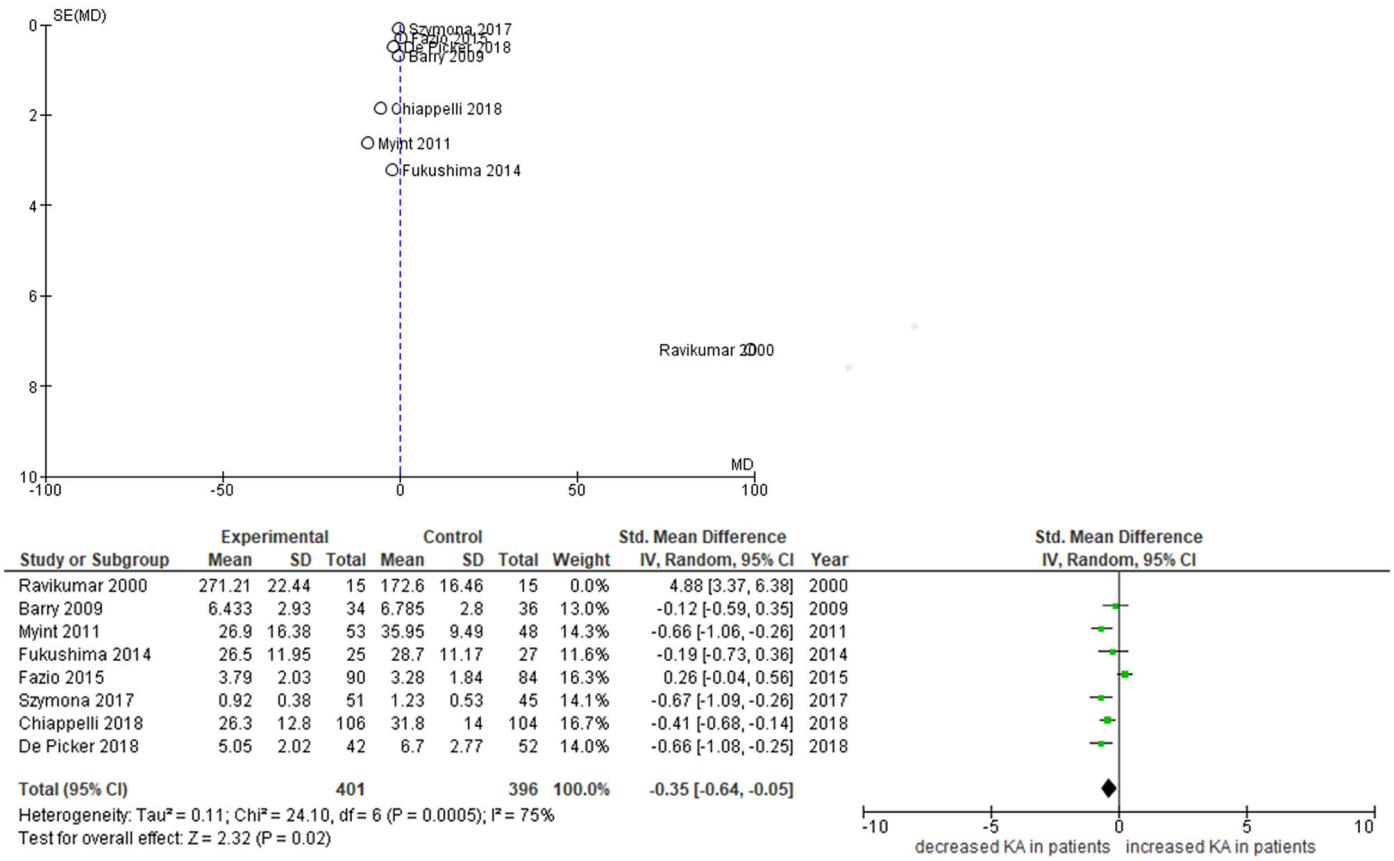
Funnel and forest plots of studies on peripheral KA levels. Funnel plot depicts standardized mean differences (SMDs) of studies on peripheral KA levels. Forest plot excludes Ravikumar et al. from analysis.

Furthermore, subgroup analyses of a recent meta-analysis by Plitman et al. of 13 studies in schizophrenia demonstrated that KA levels were increased centrally (cerebrospinal fluid and brain tissue, *n* = 7 studies) but not peripherally (*n* = 5 studies) (28). However, two studies of peripheral IDO pathway metabolites which have emerged since then suggest a relative decrease in KA even in the presence of a pro-inflammatory state (29, 30). We therefore repeated this meta-analysis using the same methods, while adding the data of the two newer studies plus our own findings. Significant study heterogeneity existed in the main analysis (*I*^2^ = 90%), with the funnel plot indicating the smallest and oldest study (31) acted as an outlier, influencing the overall result. When this study was excluded, the meta-analysis of the remaining seven studies indicated KA levels were mildly but significantly decreased in the blood of patients with schizophrenia compared to controls (standardized mean difference −0.35, *p* = 0.020) (cfr. Figure 3).

Our study has specifically looked at state-specific changes of neuroendocrine markers. Only a few other studies have longitudinally investigated IDO pathway metabolite concentrations in both arms downstream from kynurenine in psychosis patients. Myint et al. (32) studied 53 medication-free patients with schizophrenia admitted to hospital with psychotic symptoms and treated with antipsychotic medication over 6 weeks. They also identified decreased KA and KA/Kyn as trait markers, but found increased 3-HK at admission, which normalized after 6 weeks of treatment (32). Fazio et al. (33) found decreased QA and 3-HK in first- and multiple-episode schizophrenia patients, the latter of which increased significantly in first-episode patients after 1 year of treatment. KA levels were found to be increased in their study, however this result may have been confounded by a gender imbalance in this study (69% male in patients vs. 44% in controls), considering KA levels are lower in females than males (33, 34). Szymona et al. (30) analyzed blood levels of KA and 3-HK in 51 chronic schizophrenia patients during acute relapse, after 4 weeks of therapy and at remission. KA levels were significantly lower in comparison with controls throughout the study, whereas 3-HK did not differ from controls at admission and during therapy but increased at remission and correlated negatively with the improvement of negative symptoms (SANS scores) at discharge–matching our findings for PANSS negative scale scores. Finally, Wurfel et al. demonstrated reductions in serum KA and KA/QA in acutely ill inpatients with affective psychosis.

### Age and Sex Effects

The term “inflammaging” (35) has been coined to indicate significant relationships between aging and circulating concentrations of immune markers such as IL-6 and neopterin, as well as increased tryptophan breakdown in the presence of immune activation in the elderly (36). Moreover, we have recently demonstrated important age effects on microglial activity during psychosis in a subpopulation of our current sample, in whom TSPO radioligand uptake was measured using Positron Emission Tomography (37, 38).

In the current study, we observed that state and trait increases in IL1RA and IL6 became more pronounced in older patients, while state-dependent changes of QA and 3-HK were more pronounced in younger patients. Not unimportantly given the sexual dimorphism in age of onset and progression of schizophrenia, a significant interaction between cohort and sex existed in KA.

### Limitations

There are some noteworthy limitations to the present study. Firstly, the number of samples obtained was not the same in all patients. Our aim was to obtain blood samples at the earliest possible time during the acute psychotic episode. Therefore, some patients were enrolled in the study within 48 h of being admitted to hospital and before the initiation of antipsychotic medication (UMP timepoint), whereas others only entered the study at a later timepoint (Psychosis, timepoint) within the first 2 weeks of hospitalization. We therefore preferred methods of analyses which are less affected by missing or unbalanced data, such as linear mixed models. Secondly, our naturalistic study design does not allow us to differentiate to which extent changes between the different illness states can be accounted for by effects of treatment with antipsychotic medication, non-specific aspects of being hospitalized or natural illness course. Furthermore, although there is a significant reduction between baseline and follow-up PANSS total and positive subscale scores, our follow-up period of 8 weeks may still have been too short for the immune markers to normalize. A longitudinal study with longer follow-up period would be needed to monitor the evolution of state makers and to verify if the trait markers indeed remain altered in patients irrespective of their clinical course (39).

All patients in our study were started on a regimen of antipsychotic treatment during the follow-up period. Unfortunately, our data did not allow us to compare neuroimmune markers against the type and dose of antipsychotic treatment. Antipsychotics in general have been suggested to increase nitric oxide plasma levels, which would explain the sharp increase in nitrite levels in patients after antipsychotics were initiated (40). Thirdly, our findings concern peripheral measures which cannot be generalized to the central nervous system and are susceptible to confounding by factors which affect peripheral bioavailability. We repeated our analysis controlled for albumin concentration which did not alter our findings. All samples were taken in non-fasting conditions, except UMP which were usually drawn together with routine clinical sampling in early morning fasting conditions—as plasma kynurenine is typically lower in non-fasting conditions, it is unlikely this would explain our findings (41). Our IDO pathway results were very consistent despite having been generated in two different labs (University of Antwerp and University of Innsbruck) and with two different methods (LC-MS/MS vs. HPLC). In contrast, the GTP-CH1 pathway results were subject to considerable batch effects which could have nullified any biological differences in Phe, Tyr, and Neopterin.

The importance of IDO pathway metabolites is usually linked to their actions in the central nervous system. TRP, KYN, and 3-HK readily cross the blood-brain barrier while KA and QA cannot (42). Human brain kynurenines are not autonomous but are linked to, and influenced by, the peripheral IDO pathway (5). Sixty to eighty percentage of cerebral KYN—the predominant source of downstream cerebral IDO pathway metabolites—is contributed from the periphery, where the highly regulated IDO pathway accounts for ~80% of non-protein-bound Trp metabolism (43, 44). During inflammation and enhanced tryptophan breakdown, increased amounts of peripheral KYN are transported across the blood-brain barrier and become available for further downstream metabolization in astrocytes and microglia of the central nervous system. Finally, while our work has focused on IDO-initiated kynurenine metabolism, it is worth pointing out the existence of an alternative—albeit less well-studied—route catalyzed by TDO2. While TDO2 expression in mammals is mostly restricted to the liver, one study has demonstrated a 1.6-fold increase in TDO2 mRNA as well as increased density of TDO2-immunopositive astrocytes in postmortem tissue of patients with schizophrenia (45). Clearly further work is needed to elucidate the differential pathophysiological roles of IDO- vs. TDO2-mediated kynurenine metabolism in pro-inflammatory states.

### Clinical Relevance and Recommendations for Future Research

After adjustment for potential confounders and multiple testing we find that immune and neuroendocrine profiles of patients differ throughout the course of a psychotic episode. Future studies evaluating these compounds in both exploratory or interventional designs should therefore carefully select or differentiate between patients in different illness stages.

While positive symptom scores during the acute episode correlated mostly with markers of the pro-inflammatory state, IDO pathway markers were associated with negative symptom scores. Normalization of KA and 3-HK levels between admission and follow-up corresponded to a larger improvement of negative symptoms. A reverse association was found between relative improvement of SDST scores and decreased KA levels, which could represent the first evidence in humans of the preclinical findings that sudden increases in brain kynurenic acid impair cognitive flexibility (8). More comprehensive research in larger samples would be needed to further explore this relationship.

Clearly, the most unexpected finding of this study is the global downregulation of both arms of the IDO pathway in psychosis. The IDO or kynurenine pathway has been of interest to schizophrenia research because of its strong relation to the immune system as well as the fact that KA tightly controls glutamatergic and dopaminergic neurotransmission and influences behavior in animals (46). In humans, exogenous glutamate receptor antagonists induce schizophrenia-like phenomena in healthy controls.

Based on our findings, which are echoed by recently published evidence on decreased peripheral KA concentrations, a critical re-evaluation of the kynurenic acid hypothesis is needed. Alternative hypotheses to identify the origins of the IDO pathway abnormalities in schizophrenia, their relation to treatment responses and in particular the striking discrepancies between central and peripheral findings should be considered (29). Similar to a mechanism proposed in glioblastoma patients, in whom plasma concentrations of Trp, Kyn, KA, and QA were found to be decreased compared with healthy controls in the context of CNS IDO1 upregulation (47), decreased peripheral KA could be indicative of an increased demand for and transfer of Trp or Kyn through the blood–brain barrier to serve as substrate for local synthesis of KA in brain tissue. This corresponds to increased KA concentrations in the CSF of patients with schizophrenia (48). This hypothesis however does not explain why KA remains decreased throughout the illness course as trait marker while, especially in younger patients, 3-HK and QA concentrations increase post-treatment. Nor does it clarify why we found Kyn, 3-HK, and QA concentrations to correlate with TNFα concentrations in patients while KA levels did not.

Despite several decades of increased attention for kynurenines in the field of mental health research, many basic issues about their pathophysiology remain unsettled as recently extensively reviewed by Schwarcz and Stone (42). The effect of persistent up- or downregulation of kynurenine pathway metabolism in the periphery on the dynamics of blood-brain barrier transport have not yet been studied. Future work is needed to clarify the functional dynamics of IDO pathway metabolism in the CNS as well as the implications of peripheral kynurenine changes. Ideally future studies in psychosis patients should look at both kynurenine pathway arms, comparing both CSF and plasma and longitudinally following patients throughout the course of their illness.

## Conclusion

To our knowledge, this is the first study to comprehensively and longitudinally evaluate state and trait changes of IDO and GTP-CH1 pathway metabolites in parallel to immune markers in psychosis patients. Our study confirmed that the acutely psychotic (and unmedicated) state is marked by specific state increases of immune markers (IL6, IL8, TNFα, CCL2, IL1RA) and decreased Nitrite. We also demonstrated these increases in peripheral pro-inflammatory immune markers are accompanied by state-specific decreases in peripheral 3-HK and QA. Trait markers which differentiate psychosis patients from healthy controls throughout the illness course were increased CRP, CCL2, and IL1RA, and decreased KA and KA/Kyn. While PANSS positive scale scores during the acute episode correlated with pro-inflammatory immune markers, IDO pathway markers were associated with negative scale scores and normalization of KA and 3-HK levels between admission and follow-up corresponded to a larger improvement of negative symptoms. A reverse association was found between relative improvement of SDST scores and decreasing KA levels.

## Data Availability Statement

The datasets generated for this study are available on request to the corresponding author.

## Ethics Statement

The studies involving human participants were reviewed and approved by University Hospital Antwerp, Wilrijkstraat, Edegem, Belgium (main EC); supplemental approval from local Belgian ECs Emmaüs, Brothers of Charity, Spes et Fides. The patients/participants provided their written informed consent to participate in this study.

## Author Contributions

LDP, PB, MT, BS, and MM devised the project, the main conceptual ideas, and proof outline. LDP, RV, VC, DF, and HO carried out the experiments. EF verified the statistical analysis methods. LDP wrote the manuscript with support from MM, VC, and BS. All authors discussed the results and contributed to the final manuscript.

### Conflict of Interest

MT and PB are employees of Janssen Pharmaceutica N.V. and the findings of this research may affect future research lines within the company. The remaining authors declare that the research was conducted in the absence of any commercial or financial relationships that could be construed as a potential conflict of interest.
