# Supplementary material for: Immune and Neuroendocrine Trait and State Markers in Psychotic Illness: Decreased Kynurenines Marking Psychotic Exacerbations
Source: Front Immunol. 2020 Jan 17;10:2971. doi: 10.3389/fimmu.2019.02971 (PMC6978914; doi:10.3389/fimmu.2019.02971)
Supplement: Supplementary file 1 [file Data_Sheet_1.docx]

Supplementary Material

# LCMS quantitative analysis of Trp, Kyn, QA, and KA of plasma samples

# IDO pathway analytes Tryptophan (TRP), Kynurenine (Kyn), Quinolinic Acid (QA) and Kynurenic Acid (KA) were measured in citrate plasma samples using liquid chromatography–tandem mass spectrometry (LCMS) analysis at the Institute of Legal Medicine and Core Facility Metabolomics of the Medical University of Innsbruck, Austria. Samples were shipped frozen to Innsbruck where they were stored at -20°C until analysis. Samples were processed in batches of 20-30 samples. Quality control samples were added to each batch as quality control samples. The order in which the samples were processed was prespecified to make sure all samples belonging to the same subjects were in the same batch, and each batch contained a similar number of patient and control samples.

## Chemicals and reagents

# TRP, KYN, KA, QA, formic acid, aqueous ammonium hydroxide solution (25 %), and activated charcoal (Darco, 100 mesh), acetonitrile, and water were purchased from Sigma Aldrich (St. Louis, MO, USA). TRP-d5, KYNA-d5, and QUIN-d3 were obtained from Toronto Research Chemicals (North York, Ontario, Canada).

## Surrogate matrix

# Surrogate matrix for preparing reference standards was obtained from treating pooled drug-free human plasma with activated charcoal.

## Preparation of standard and quality control samples

# Individual stock solutions containing 1.0 mg/ml of each analyte or the internal standards (IS) were prepared in water/acetonitrile (50:50, v/v) and stored at -20 °C. Working solution mixtures containing TRP, KYN, KYNA, and QUIN at concentrations 0.2-100 µg/ml, 0.02-10 µg/ml, 1-500 ng/ml, and 0.02-10 µg/ml, respectively, were prepared by dilution of the stock solutions with water. The IS working solution contained 121 ng/ml KYNA-d5, 1210 ng/ml QUIN-d3 as well as 60.5 µg/ml TRP-d5, and was prepared by dilutions of the stock solutions with water. Calibration samples were prepared by spiking 50 µl of charcoal purified plasma with 5 µl of IS working solution and 25 µl of working calibrator solutions resulting in calibration levels 0.2, 0.5, 1.0, 5.0, 10, 50 µg/ml for TRP, 20, 50, 100, 500, 1000, 5000 ng/ml for KYN and QUIN, as well as 1.0, 2.5, 5.0, 25, 50, 250 ng/ml for KYNA.

## Quality control samples

# Plasma samples were used as quality controls (serum B, serum D), and they were kindly donated by the blood bank of the Medical University of Innsbruck (Austria). They were stored at -20 °C.

## Sample preparation

# Sample preparation included protein precipitation with acetonitrile. Therefore, 50 µl plasma sample were mixed with 5.0 µl IS working mixture, 25 µl water, and 70 µl acetonitrile and sonicated for 5 min. Next, the mixture was centrifuged at 4500 rpm for 5 min at room temperature and 100 µl of the supernatant were transferred to a glass autosampler vial and 5 µl aqueous ammonium hydroxide solution (25%) was added. Samples were analyzed within 24 hours after extraction by injecting 5.0 μl into the LC/MS/MS system. Extracts were stored at 8°C in the autosampler system.

## Liquid chromatography–tandem mass spectrometry

# The chromatographic system consisted of an Ekspert nanoLC 425 pump (Eksigent, Dublin, CA, USA) and an Ekspert nanoLC 400 autosampler (Eksigent) equipped with a 10 µl injection loop. The autosampler tray temperature was set to 8 °C. Chromatographic separations were accomplished on a Luna NH2 column (150 x 1.00 mm, 3 µm, 100 A, Phenomenex, Torrance, CA, USA) protected by a guard column (SecurityGuard equipped with an Luna NH2 4 x 2.0 mm cartridge, Phenomenex) by applying a linear gradient of 50-5% acetonitrile in aqueous ammonium acetate solution (5 mM, pH 9.5) within 6 min. After flushing the column with 5% acetonitrile for two minutes, the column was re-equilibrated with 5 mM ammonium acetate (pH 9.5) containing 50 % acetonitrile for 20 min. The flow rate was set to 35 µl/min and the total run time was 28.5 min. The column temperature was set to 30 °C applying a column oven (Eksigent). Mass spectrometric analysis was performed on a quadrupole-quadrupole time-of-flight instrument (TripleTOF5600+, Sciex). The mass spectrometer was equipped with a DuoSpray ion source, and was operated in the negative electrospray ionization mode. The spray voltage was set to -4.5 kV. Gas flows of 30 arbitrary units were employed for the nebulizer gas the turbo gas. The temperature of the turbo gas was adjusted to 300 °C. For MS/MS experiments, Q1 was set to unit resolution and MS/MS spectra were recorded at m/z 50-300 in high sensitivity mode. Compound-specific HR-MRM parameters are summarized in Table 1. The instrument was operated at a mass resolution of ∼15,000 for MS/MS and automatically recalibrated every five sample injections using APCI negative calibration solution delivered via an integrated calibration delivery system (AB Sciex). Data acquisition was performed on a personal computer with Analyst TF 1.6 software and quantitative data analysis was performed with MultiQuant 2.1.1 software (both Sciex).

# Supplementary Figures and Tables

## Supplementary Figures


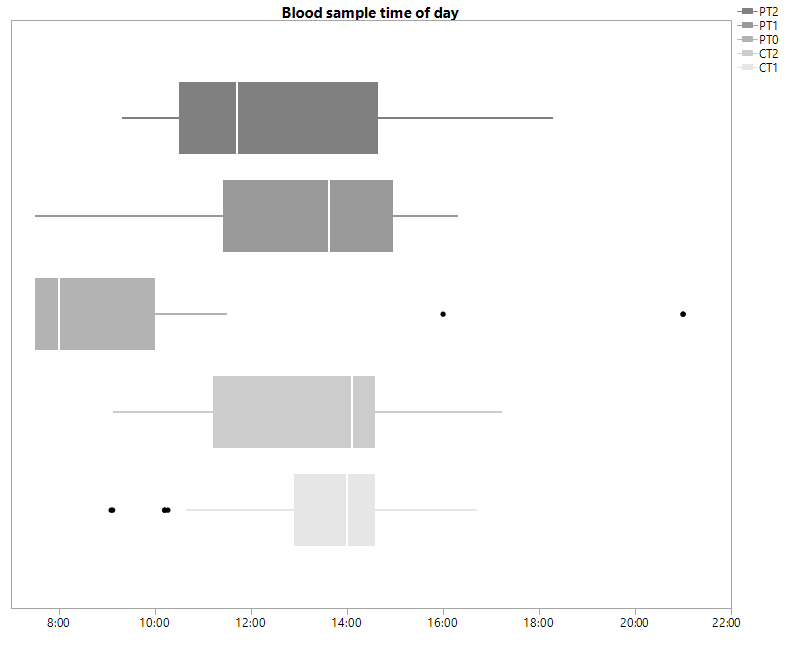


**Supplementary Figure 1: Timing of blood draws**

Legend: CT1: first blood sample in controls; CT2: second blood sample in controls; PT0: UMP sample in patients; PT1: Psychosis sample in patients; PT2: Follow-up sample in patients. PT0 samples were drawn together with routine clinical lab draws during early morning rounds and therefore drawn significantly earlier compared to the other samples. Blood draws at T1-T2 were matched as closely as possible in each individual subject; matched pairs T-test no significant difference between T1 and T2 (t-1.8, p.071) and no difference between cohorts (within pairs F0.02; among pairs F1.09).

## Supplementary Tables

Supplementary Table 1: Longitudinal linear mixed model analyses
Outcomes of the final longitudinal linear mixed models, including relevant confounders.

| **Model** | | **State = acute psychotic episode (UMP + Psychosis timepoints)** | | **State = UMP timepoint** | |
| --- | --- | --- | --- | --- | --- |
| *Marker* | *Term* | *F-ratio* | *p-value* | *F-ratio* | *p-value* |
| **IL6** | Cohort  State[Cohort]  Age | 4.02  3.85  7.45 | .048  .052  .008* | 10.34  21.03  6.41 | .002*  <.001*  .013* |
| **IL8** | Cohort  State[Cohort] | 4.94  3.25 | .028  .074 | 9.23  15.88 | .003*  <.001* |
| **IL1RA** | Cohort  State[Cohort]  BMI | 8.30  8.33  14.15 | .005*  .005*  <.001* | 14.82  11.20  12.44 | <.001*  .001*  .001* |
| **IL1B** | Cohort*Age  State[Cohort] | 7.96  0.71 | .006*  .402 | 6.52  0.81 | .012*  .369 |
| **TNFα** | Cohort  State[Cohort] | 2.46  0.17 | .121  .679 | 3.38  8.81 | .070  .004* |
| **CRP** | Cohort  State[Cohort]  BMI | 5.96  0.03  24.17 | .017*  .866  <.001* | 7.51  2.02  22.65 | .007*  .158  <.001* |
| **CCL2** | Cohort  State[Cohort] | 10.45  0.01 | .002*  .969 | 12.92  8.68 | <.001*  .004* |
| **Kyn** | Cohort  State[Cohort] | 2.09  2.39 | .151  .125 | 2.23  0.18 | 0.139  0.672 |
| **Kyn/Trp** | Cohort*Age  State[Cohort] | 8.94  5.04 | .004*  .027 | 9.04  0.01 | .003*  .952 |
| **KA** | Cohort*Sex  State[Cohort] | 17.39  0.47 | <.001*  .050 | 17.50  0.13 | <.001*  .721 |
| **KA/Kyn** | Cohort*Sex  State[Cohort] | 13.64  0.56 | <.001*  .454 | 13.56  0.12 | <.001*  .732 |
| **QA** | Cohort  State[Cohort]  BMI | 3.36  16.07  5.39 | 0.07  <.001*  .022* | 7.50  6.34  5.42 | .007*  .013*  .022* |
| **QA/KA** | Cohort  State[Cohort] | 3.99  4.50 | .048  .035 | 1.51  6.63 | .222  .011* |
| **3-HK** | Cohort  State[Cohort]  Batch | 2.55  12.81  22.96 | .114  .001*  <.001* | 5.93  5.39  22.11 | .017*  .022*  <.001* |
| **Nitrite** | Cohort  State[Cohort]  Sex  Batch | 3.85  11.76  12.07  0.91 | .053  .001*  .001*  .342 | 0.01  42.36  13.35  0.79 | .971  <.001*  <.001*  .374 |
| **Phe/Tyr** | Cohort  State[Cohort]  Batch | .085  .144  260.33 | .359  .705  <.001* | 1.49  2.95  268.71 | .224  .088  <.001* |
| **Neopterin** | Cohort  State[Cohort]  Batch | .262  .832  14.26 | .610  .363  <.001* | .223  .187  15.05 | .638  .666  <.001* |

Supplementary Table 2: Correlations between immune marker concentrations and IDO pathway metabolites.

| **Patients** |  | Kyn | Kyn/Trp | QA | QA/KA | 3-HK | KA | KA/Kyn |
| --- | --- | --- | --- | --- | --- | --- | --- | --- |
| TNFα (Admission) | r | .310 | .461 | .481 | .518 | .210 | -.193 | -.330 |
|  | p-value | .151 | .027 | .020 | .011 | .335 | .377 | .124 |
| TNFα (Psychosis) | r | .466 | .361 | .451 | .446 | .362 | -.153 | -.392 |
|  | p-value | .016 | .070 | .021 | .023 | .069 | .456 | .048 |
| TNFα  (Follow-up) | r | .509 | .556 | .432 | .349 | .556 | .122 | -.241 |
|  | p-value | .018 | .009 | .051 | .121 | .009 | .599 | .292 |
| **Controls** |  | Kyn | Kyn/Trp | QA | QA/KA | 3-HK | KA | KA/Kyn |
| CRP | r | .268 | .283 | .516 | .275 | .389 | .080 | -0.08 |
|  | p-value | .004 | .003 | <.001 | .004 | <.001 | .404 | .391 |
| IL1RA | r | .253 | .329 | .416 | .278 | .206 | .016 | -.097 |
|  | p-value | .010 | <.001 | <.001 | .005 | .037 | .872 | .328 |
